# Effects of galanin receptor 2 and receptor 3 knockout in mouse models of acute seizures

**DOI:** 10.1111/epi.14573

**Published:** 2018-10-08

**Authors:** Meinrad Drexel, Felix Locker, Barbara Kofler, Günther Sperk

**Affiliations:** ^1^ Department of Pharmacology Medical University of Innsbruck Innsbruck Austria; ^2^ Laura Bassi Centre of Expertise‐THERAPEP Research Program for Receptor Biochemistry and Tumor Metabolism Department of Pediatrics Paracelsus Medical University Salzburg Austria

**Keywords:** GAL2‐R, GAL3‐R, hippocampus, kainic acid, pentylenetetrazole, seizure protection

## Abstract

There exists solid evidence that endogenous galanin and galanin agonists exert anticonvulsive actions mediated both by galanin 1 receptor (GAL1‐R) and galanin 2 receptor (GAL2‐R). We have now investigated whether depletion of the recently identified third galanin receptor, GAL3‐R, and that of GAL2‐R, alters the threshold to the systemically applied γ‐aminobutyric acid (GABA) antagonist pentylenetetrazole (PTZ) or to intrahippocampally administered kainic acid (KA). In neither model, GAL3‐KO mice differed in their latency to the first seizure, mean seizure duration, total number of seizures, or time spent in seizures compared to wild‐type controls. In addition, consistent with previous data, the response to PTZ was not altered in GAL2‐KO mice. In contrast, intrahippocampal KA resulted in a significantly increased number of seizures and time spent in seizures in GAL2‐KO mice, although the latency to the first seizure and the duration of individual seizures was not altered. These results are consistent with the previous data showing that GAL2‐R knockdown does not affect the number of perforant path stimulations required for initiating status epilepticus but significantly increases the seizure severity during the ongoing status. In conclusion, our data support a specific role of GAL2‐R but not of GAL3‐R in mediating the anticonvulsive actions of endogenous galanin.

## 1 INTRODUCTION

There is solid evidence that endogenous galanin and galanin agonists exert anticonvulsive actions in animal models of acute and chronic seizures,^1^, ^2^ and local injection of a viral vector overexpressing galanin reduces the number and severity of seizures induced by focal intrahippocampal injection of kainic acid (KA).^3^ Galanin‐KO mice exert a lowered threshold to seizures,^4^ and galanin antagonists exert proconvulsant effects,^5^ indicating a role of endogenous galanin in protecting from the expression of epileptic seizures. The anticonvulsive action of galanin may be mediated differently by galanin 1 receptor (GAL1‐R) and galanin 2 receptor (GAL2‐R). Thus deletion of GAL1‐R resulted in increased seizure susceptibility, indicating an important role of GAL1‐R in mediating the anticonvulsive actions of endogenous galanin.^4^ In contrast, GAL2‐KO mice did not show altered seizure susceptibility to pentylenetetrazole (PTZ) or flurothyl^6^ although 50% downregulation of GAL2‐R in the dentate gyrus increased the time spent in seizures in a model of self‐sustaining status epilepticus, and the selective GAL2‐R agonist Ala^2^‐galanin antagonized these effects.^7^ In addition, the GAL2‐R/GAL3‐R agonist galanin (2‐11) prevents electrical kindling,^8^ further supporting a role for GAL2‐R in the endogenous anticonvulsive action of galanin.

A possible role of GAL3‐R in the anticonvulsive action of galanin has not been investigated so far. In our present experiments, we investigated the effect of GAL3‐R and GAL2‐R depletion in 2 mouse models of acute seizures: (1) In seizures induced by intraperitoneal application of the GABA antagonist PTZ and (2) in seizures induced by intrahippocampal application of the excitatory amino acid receptor agonist KA.

## 2 METHODS

### 2.1 Mice

We used 10‐ to 14‐week‐old male mice (GAL2‐KO, GAL3‐KO) backcrossed on C57BL/6 background and C57BL/6 wild‐type (WT) mice as controls.^6^, ^9^ Experiments were conducted according to European Community laws and were approved by the Committee for Animal Protection of the Austrian Ministry of Science. Mice were housed in groups of 3‐5 in single‐ventilated cages under standard laboratory conditions (12 hour/12 hour light/dark cycle) and had access to food and water ad libitum.

### 2.2 Surgery

Implantation of telemetric electroencephalography (EEG) transmitters (TA10EA‐F20, Data Sciences International [DSI], Arden Hills, Minnesota) was performed as described previously.^10^ Epidural EEG electrodes were set at the following coordinates, in mm: 2.0 lateral and 2.8 posterior from bregma; a reference electrode was placed at the right side, 1.5 lateral and 2.0 posterior from the bregma. For intrahippocampal injections, an epidural guide cannula (polyimide‐coated silica capillary tubing; 350 μm diameter; Polymicro Technologies, Molex, Walldorf, Germany) was set above the left dorsal hippocampus through a previously drilled hole (1.0 mm lateral, 1.7 mm posterior) and fixed to the skull with dental cement. The guide cannula was closed by inserting a stainless steel wire dummy. After surgery, mice were single‐housed for EEG and video monitoring as described previously.^11^

### 2.3 Pentylenetetrazole (PTZ) model

One week after transmitter implantation, mice were injected intraperitoneally (ip) with a threshold dose of PTZ (45 mg/kg in saline, pH 7.4; Sigma‐Aldrich, Vienna, Austria) and EEG traces were recorded.

### 2.4 Induction of focal‐onset seizures by intrahippocampal kainic acid (KA) injection

Two weeks after surgery, mice were anesthetized briefly with 3% sevoflurane and injected intrahippocampally with KA (7 ng in 500 nL phosphate‐buffered saline [PBS], pH 7.4, injection speed 500 nL/min; pump: Ascent Scientific, North Somerset, UK). The injection cannula (polyimide‐coated silica capillary tubing: 150 μm outer diameter; TSP075150, Polymicro Technologies) was attached to a 5 μL Hamilton syringe using fine‐bore polythene tubing (800/100/100; Smiths Medical International, Kent, UK) and connected to a precision syringe pump (Nexus 3000 by Chemyx, Science Products, Hofheim, Germany). After injection, the cannula was left in place for 1 minute before it was slowly retracted, and the mice were quickly transferred to their cages for EEG recording. Under these conditions, KA induced acute seizures in 100% of mice and EEG activity returned to baseline after 1.5‐2 hours.

### 2.5 EEG and behavioral analysis of seizures

EEG activity was recorded continuously at a sampling rate of 1000 Hz with no a priori filter cutoff using an EEG recording system (Ponemah Physiology Platform 6.30, DSI).^10^ EEG recordings were analyzed using the same software. Seizures were defined as EEG segments with continuous high‐frequency activity and an amplitude of at least 2 times baseline amplitude and a duration of at least 10 seconds. Latency to first seizure, seizure numbers, mean seizure duration, and total time spent in seizures were analyzed. The behavior of the mice was video monitored^10^ and seizures were rated as described.^11^

### 2.6 Statistical analysis

Statistical analyses were performed using GraphPad Prism 5 (GraphPad, La Jolla, California). Fisher's exact test was used for analysis of the effect of threshold dose PTZ in galanin receptor KO mice and WT littermates. One‐way analysis of variance (ANOVA) with Bonferroni's multiple comparison post hoc test was used for group comparisons. A *P* value <0.05 was considered statistically significant.

## 3 RESULTS

### 3.1 Effect of GAL2‐R or GAL3‐R deletion on PTZ‐induced seizures

**Figure 1:**
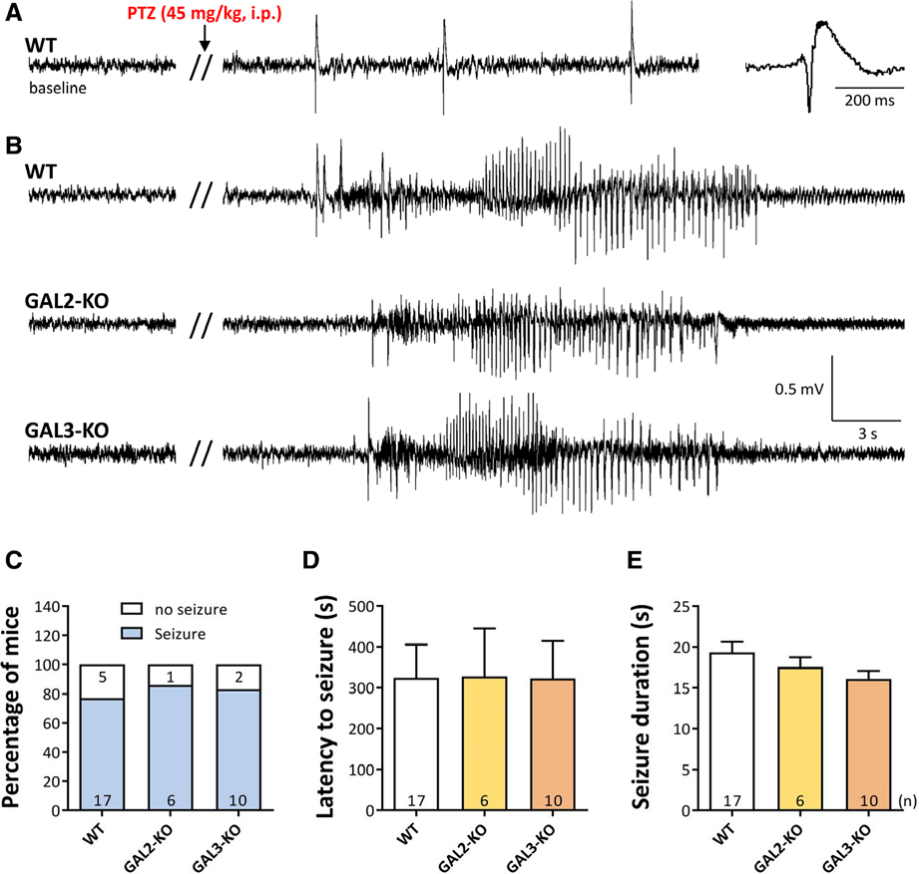
Effect of GAL2‐R and GAL3‐R knockout on acute PTZ‐induced seizures in mice. A and B, Representative EEG traces after intraperitoneal injection of a threshold dose of PTZ (45 mg/kg). About 10%‐20% of the mice revealed only spike‐wave discharges but no seizure during the 60 min observation period (A). In B, typical EEG traces of motor seizures in wild‐type (WT), GAL2‐KO, and a GAL3‐KO mouse are shown, respectively. About 80%‐90% of mice expressed a single generalized motor seizure. In C, the percentage of mice developing an EEG seizure; in D, the mean latency to this seizure; and in E, the mean duration of the seizure is shown. Error bars represent standard errors of the mean

Upon intraperitoneal PTZ injection, 77% of WT, 84% of GAL2‐KO, and 86% of GAL3‐KO mice showed a single generalized motor seizure recorded behaviorally and on EEG (Figure 1B,C; not significantly different). Mice that failed to develop a seizure presented intermittent spike‐wave discharges (Figure 1A). Furthermore, neither the *latency to the seizure* (Figure 1D) nor the *mean seizure duration* (Figure 1E) differed significantly between any of the 3 experimental groups.

### 3.2 Acute seizures induced by intrahippocampal injection of KA

**Figure 2:**
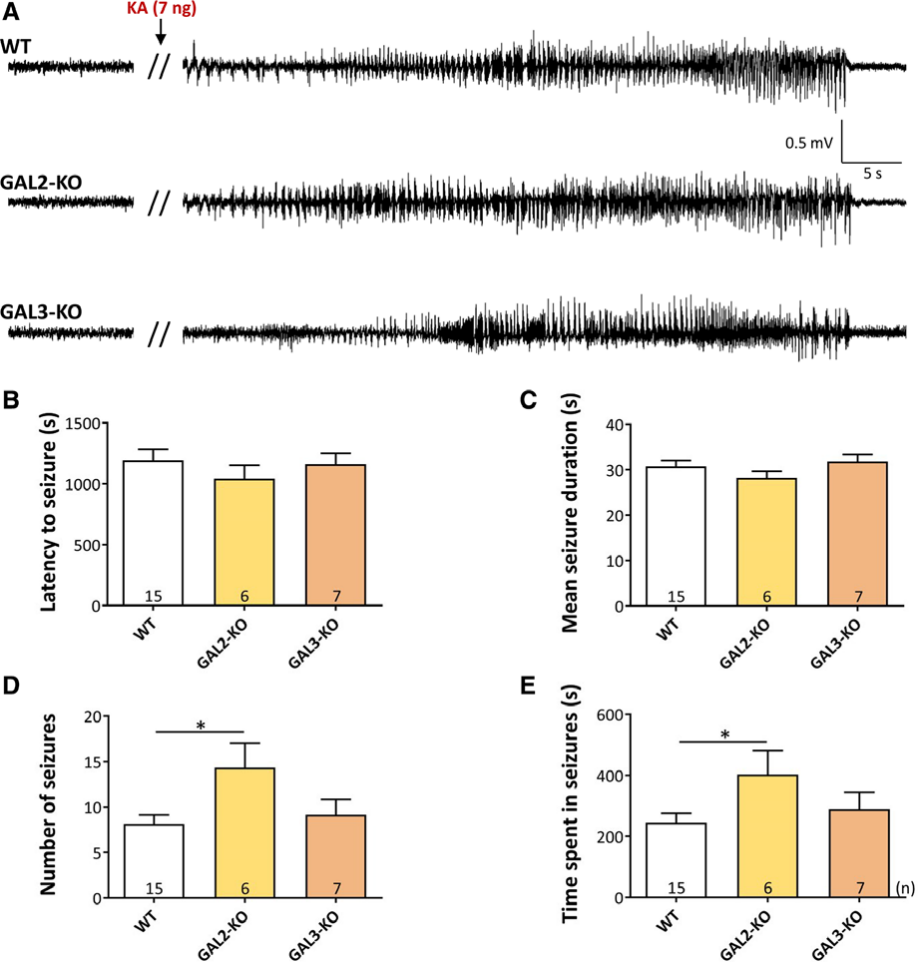
Acute seizures induced by intrahippocampal injection of KA. A, Representative EEG traces showing the baseline EEG after termination of sevoflurane anesthesia and one acute seizure in WT, GAL2‐KO, and GAL3‐KO mice. B, Mean latency to the first seizure; C, Mean seizure duration; D, Mean number of seizures; and E, Time spent in seizures. The error bars represent standard errors of the mean. *P <* *0.05 in the Bonferroni multiple comparison test after ANOVA

Intrahippocampal injection of KA was performed through a previously implanted guide cannula under superficial sevoflurane anesthesia. After KA injection and termination of sevoflurane anesthesia, the transiently reduced EEG activity rapidly (210 ± 7.7 seconds [Values represent mean ± standard error of the mean (SEM)]) returned to baseline activity. After 15‐25 minutes, all mice developed the first of 3‐24 seizures on EEG (Figure 2A). All seizures recorded on EEG corresponded to full generalized motor seizures (rating 3‐4^11^). We observed no differences between WT controls and GAL2‐KO, or between WT and GAL3‐KO mice in their *latency to first seizure* (Figure 2B). In addition, the *mean seizure duration* did not differ significantly between WT and GAL2‐KO or GAL3‐KO mice (Figure 2C). However, GAL2‐KO mice, but not GAL3‐KO, displayed a significantly increased *number of seizures* compared to WT controls (Figure 2D; WT vs GAL2‐KO: *t *=* *2.742, *P *<* *0.05). Accordingly, we observed for GAL2‐KO (but not GAL3‐KO) mice also a significantly increased *time spent in seizures* (Figure 2E; WT vs GAL2‐KO: *t *=* *2.291, *P *<* *0.05).

## 4 DISCUSSION

The primary goal of our experiments was to examine whether GAL3‐R participates in the anticonvulsive action of endogenous galanin and to compare the effects of GAL3‐R against GAL2‐R deletion in mice. We tested possible differences in the seizure threshold after acute seizures induced either by intraperitoneal PTZ, or by intrahippocampal KA. We did not observe any difference between GAL3‐KO and WT mice in either model of acute seizures. In addition, GAL2‐R deletion did not change the response to PTZ but resulted in a significantly increased seizure number and total time spent in seizures after intrahippocampal KA. However, neither the latency to the first seizure nor the mean seizure duration was altered in GAL2‐KO mice after KA.

Taken together, the data indicate that endogenous galanin, besides acting through GAL1‐R,^4^ may have a protective function mediated by GAL2‐R but not GAL3‐R. Thus, also the GAL2‐R preferring agonist NAX 810‐2 protects from kindled seizures.^12^ The anticonvulsive action of GAL2‐R, however, seems to depend on the anatomic site and the mode of seizure initiation. Whereas PTZ induces seizures at multiple sites of the brain and spinal cord by inhibiting GABA_A_ receptors, intrahippocampal application of KA acts by activation of ?‐amino‐3‐hydroxy‐5‐methyl‐4‐isoxazole propionic acid (AMPA) and KA receptors in the hippocampus. GAL2‐R seems to modify only the response to intrahippocampal KA but not that to PTZ or flurothyl (our present results and those by Gottsch et al^6^). In contrast, stimulation of GAL1‐R inhibits seizure induction by PTZ. Thus, the galanin agonist galnon prevents rats from PTZ‐induced seizures, an effect that is abolished by pretreatment with a GAL1‐R antisense probe.^5^ In fact, GAL1‐R is mediating anticonvulsive actions of endogenous and exogenous galanin in a variety of epilepsy models including kindling and status epilepticus induced by chemoconvulsants or perforant path stimulation.^13^ In line with these findings, galmic, a specific nonpeptide agonist of GAL1‐R, protects from status epilepticus induced by repeated perforant path stimulation.^14^

Galanin is contained in nerve terminals of glutamate, noradrenaline, and acetylcholine neurons.^2^, ^15^ Through presynaptic receptors, galanin may inhibit release of glutamate in the hippocampus (particularly in models of perforant path stimulation or KA‐induced seizures)^16^ or that of noradrenaline and acetylcholine at respective projections, for example, to the hippocampus or amygdala resulting in seizure protection.^4^ Thus, galanin is especially potent in models involving stimulation of the cholinergic systems by Li‐pilocarpine.^4^

Our present experiments demonstrate that GAL2‐R deletion augments seizures induced by intrahippocampal KA. Although not directly comparable to status epilepticus induced by systemic KA, also in this model moderate activation of hippocampal circuitries for about 15‐25 minutes leads to the development of seizures and allows endogenous galanin to become active. Strikingly, the response of the GAL2‐KO mice to intrahippocampal KA was graded. Whereas their latency to the first seizure and the mean seizure duration were not altered, they expressed a significantly higher number of seizures and, therefore, total time spent in seizures. This is reminiscent of the effect of partial GAL2‐R messenger RNA (mRNA) knockdown on status epilepticus induced by perforant path stimulation.^7^ Similarly, this treatment did not affect the number of perforant path stimulations required for initiating the status but significantly increased the severity of seizures during the ongoing status. Considering a mechanism of endogenous galanin suppressing transmitter release in projection neurons through presynaptic GAL2‐R,^4^, ^15^ it is possible that galanin may not suffice to suppress the massive neurotransmitter release during seizure onset^11^ but may be able to protect from the more moderate transmitter release during late spontaneous seizures.

On a molecular basis, the anticonvulsive actions of GAL1‐R may involve activation of a G_i_ protein–mediated inhibition of cyclic AMP (cAMP) synthesis and the opening of G protein–regulated inward rectifying K^+^ channels (GIRK) resulting in inhibition of transmitter release.^17^ The role of GAL2‐R is more complicated and involves potential proconvulsive and anticonvulsive mechanisms.^4^, ^7^ GAL2‐R are coupled to G_q/11_‐activating phospholipase C, inositol trisphosphate production, and mobilization of intracellular Ca^2+^, and therefore, are potentially proconvulsive. During kindling, however, these mechanisms seem to be masked and overruled by G_i_‐related (pertussis toxin–sensitive and GIRK‐independent) mechanisms mediating the anticonvulsive actions of GAL2‐R.^8^ GAL2‐R reduces Ca^2+^ influx through L‐type voltage‐gated Ca^2+^ channels, presumably inhibiting transmitter release,^18^ and galanin inhibits glutamate release by activating ATP‐sensitive K^+^ channels in dentate granule cells,^4^, ^16^ likely also mediated by G_o2_ protein coupled to GAL2‐R.^19^

In both of our experimental models, GAL3‐R deletion did not affect the seizure threshold. This was somewhat unexpected as GAL3‐R couples to a G_i/o_‐type G protein, activating inward rectifying K^+^ channels and therefore may also inhibit transmitter release (like GAL1‐R and GAL2‐R). Although GAL3‐R expression is restricted primarily to the hypothalamic areas, it is also expressed in the hippocampus and amygdala^2^, ^20^ where GAL3‐R deletion has profound effects on the emotional behavior of mice.^9^ Both brain areas are involved in the generation and spread of limbic seizures. On the other hand, it is possible that GAL3‐R may not be expressed sufficiently in these brain areas to become effective or may not be located, for example, at presynaptic terminals for allowing suppression of seizure initiation or generalization.

## DISCLOSURE

The authors declare no competing financial interests. We confirm that we have read the Journal's position on issues involved in ethical publication and affirm that this report is consistent with those guidelines.
